# Supplementary material for: Transcriptional Profiling and Biological Pathway(s) Analysis of Type 2 Diabetes Mellitus in a Pakistani Population
Source: Int J Environ Res Public Health. 2020 Aug 13;17(16):5866. doi: 10.3390/ijerph17165866 (PMC7460550; doi:10.3390/ijerph17165866)
Supplement: Supplementary file 1 [file ijerph-17-05866-s001.zip › Supplemental Table S2.docx]

| **Population Variables** | **(***in* **T2DM Subjects, n=15)** |
| --- | --- |
|  |  |
| Age (in years): | 56.47 ± 12.2 |
| Sex |  |
| *Male* | 46.67% |
| *Female* | 53.33% |
| Literacy |  |
| *Literate* | 33.33% |
| *Illiterate* | 66.67% |
| Locality |  |
| *Urban* | 66.67% |
| *Rural* | 33.33% |
| Industrial Area | |
| *Yes* | 13.33% |
| *No* | 86.67% |
| Medication |  |
| *Yes* | 73.33% |
| *No* | 26.67% |
| Hypertension |  |
| *Yes* | 0.40% |
| *No* | 0.60% |
| History of T2DM | |
| *Yes* | 73.33% |
| *No* | 26.67% |
| Smoking |  |
| *Yes* | 33.33% |
| *No* | 66.67% |
| HbA1c (%) | 10.8 ± 2.48 |
| Plasma Glucose (mg/dL) | 317 ± 99.38 |
| BMI | 22.73 ± 2.59 |
|  |  |

**Supplemental Table 2.**  Demographic and clinical data of the small population that underwent pilot gene validation studies.

Data are in ± SD if not otherwise mentioned
